# Uncharted territory: retinal vasculitis and cryoglobulinemia in Behçet’s disease – a case report

**DOI:** 10.3389/fimmu.2025.1533595

**Published:** 2025-07-04

**Authors:** Yadan Zou, Kun Yang, Xiaoying Zhang, Sheng-Guang Li

**Affiliations:** ^1^ Department of Rheumatology and Immunology, Peking University International Hospital, Beijing, China; ^2^ Second Department of Clinical Medicine, Shanxi Medical University, Taiyuan, China; ^3^ Department of Pathology, Peking University International Hospital, Beijing, China

**Keywords:** Behçet’s disease, mixed cryoglobulinemia, hepatitis C virus, retinal vasculitis, systemic vasculitis, immunosuppressive therapy, case report

## Abstract

Behçet’s disease (BD) is a systemic inflammatory disorder characterized by recurrent oral and genital ulcers, skin lesions, and ocular involvement, often presenting with retinal vasculitis as a severe complication. Although mixed cryoglobulinemia, typically associated with hepatitis C virus (HCV) infection, is well-documented in other autoimmune diseases, its coexistence with BD is exceedingly rare. This report details the case of a 56-year-old male presenting with BD complicated by HCV-related mixed cryoglobulinemia, manifesting as retinal vasculitis, purpuric skin lesions, and systemic vasculitis. Despite initial corticosteroid treatment, the patient required escalated therapy, including immunosuppressants and antiviral agents, to achieve disease stability. This case underscores the need for a multidisciplinary approach in managing BD with secondary cryoglobulinemia and highlights the complex interplay between autoimmune and viral-induced vasculitis. Our findings contribute to the literature by documenting a rare presentation of BD and providing insights into comprehensive treatment strategies for similar cases.

## Introduction

Behçet’s disease (BD) is a multisystem inflammatory disorder primarily characterized by recurrent oral and genital ulcers, skin lesions, and ocular involvement. It is classified as a variable-vessel vasculitis, with a unique tendency to affect both arteries and veins of all sizes, making its manifestations complex and often severe. Among these, retinal vasculitis, particularly in the form of frosted branch angiitis, represents a serious complication that can lead to permanent vision loss if not promptly treated. The condition’s pathology is often linked to immune dysregulation, with HLA-B51 being a known genetic marker associated with increased disease severity and risk of ocular involvement (1–3).

Cryoglobulinemia, defined by the presence of circulating proteins that precipitate at low temperatures, is primarily associated with hepatitis C virus (HCV) infection and autoimmune diseases like systemic lupus erythematosus and Sjögren’s syndrome. However, its occurrence in Behçet’s disease is extremely rare, with only a handful of documented cases since the late 1970s (4, 5). Cryoglobulinemia can lead to systemic vasculitis, manifesting as purpura, arthralgia, and potentially life-threatening organ involvement.

The coexistence of cryoglobulinemia and Behçet’s disease is particularly challenging, as it combines the vasculitic processes of both disorders, potentially exacerbating vascular and systemic inflammation. Herein, we present a rare case of Behçet’s disease complicated by HCV-related mixed cryoglobulinemia, manifesting as retinal vasculitis and purpuric skin lesions. This report aims to explore the complexities of such co-morbidity, drawing upon recent literature to discuss optimal diagnostic and therapeutic strategies.

## Case presentation

A 56-year-old male was admitted with a seven-day history of fever, polyarticular pain, blurred vision, and a purpuric rash on his lower limbs. His symptoms began with fever, following pain in the throat, jaw and left ear canal accompanied by tinnitus. The fever fluctuated between 37.5°C and 39.9°C and was not accompanied by cough, sputum, urinary frequency, or diarrhea. Two days later, he developed joint pain, initially affecting both knees, hips and heels, with significant swelling in the right knee and heel, leading to difficulty walking and turning in bed. Soon after, he experienced sudden visual blurring and diplopia, with marked vision loss and symmetrical, densely distributed purpuric rashes and bruises on his limbs, especially the lower extremities. During this period, multiple acneiform pustules appeared on his scalp, and the fever was temporarily alleviated with ibuprofen.

Due to progressive visual deterioration, the patient underwent ophthalmologic evaluation at another hospital on May 8, 2022, where fundus fluorescein angiography revealed retinal vascular occlusion. He was treated with alprostadil (10 µg daily), compound anisodine injections, and oral aspirin (100 mg daily), with no notable improvement in vision. He was transferred to our hospital and admitted on May 13, 2022. During the illness, he reported no genital ulcers, cough, sputum, abdominal pain, urinary symptoms, photosensitivity, Raynaud’s phenomenon, dry eyes, or hoarseness. Bowel habits and weight remained stable.

His medical history was significant for a ventricular septal defect repair with pulmonary hypertension 33 years prior. He reported a 27-year history of recurrent, painful oral ulcers, initially appearing as red painful spots that ruptured to form white-coated lesions, widely distributed on the buccal mucosa, tongue, and gums. These ulcers recurred more than ten times yearly, each lasting 7–10 days and healing without scarring. He occasionally used mouthwashes and Chinese medicine, which provided temporary symptom relief but did not reduce ulcer frequency. The patient also had a history of anal fistula surgery performed 22 years prior. Throughout the long-term disease course, he reported no abdominal pain, diarrhea, or weight loss, clinically ruling out Crohn’s disease. Additionally, he was diagnosed with diabetes 17 years ago, managed with insulin but without regular blood sugar monitoring. Family history was notable for diabetes in his father and seven siblings, with his father deceased from cerebrovascular disease. Additionally, he sustained a brain injury requiring surgery 12 years prior, with a history of blood transfusion. Over the past four years, he experienced progressive hearing loss, which developed into bilateral tinnitus a year ago, and recent pain in both shoulders and the right elbow with occasional swelling.

On admission, his temperature was 37.4°C, pulse and respiratory rate were normal, and blood pressure was 142/86 mmHg. Examination revealed multiple acne-like pustules on the scalp and occipital area, some with tenderness. Purpuric rashes were present on both lower limbs, and a 3 cm irregular bruise was noted on the right buttock. Multiple oral ulcers were visible on the buccal mucosa, and an enlarged, tender right submandibular lymph node was palpated. Cardiac examination revealed a regular rhythm, accentuated P2, and diastolic murmurs over the mitral and tricuspid valves. Examination of the musculoskeletal system showed swelling and tenderness in both knees and the right elbow. Muscle strength was preserved in all limbs, and there were no pathological reflexes or signs of meningeal irritation.

Peripheral blood tests showed a white blood cell count of 7.16 x 10^9/L, hemoglobin level of 140 g/L, and platelet count of 118 x 10^9/L. Liver and kidney function, blood glucose, and lipid levels were within normal limits. Inflammatory markers included an erythrocyte sedimentation rate of 6 mm/h, C-reactive protein of 5.31 mg/L, interleukin-6 of 48.93 pg/mL, and procalcitonin of 0.196 ng/mL. Tests for tuberculosis, TORCH infections, respiratory viruses, and COVID-19 were negative. Autoimmune serologies were unremarkable, with negative rheumatoid factor, anti-cyclic citrullinated peptide, endothelial cell antibodies, anti-neutrophil cytoplasmic antibodies, and HLA-B27. However, antinuclear antibody was positive (1:160, cytoplasmic), and both HLA-B51 and cryoglobulins were detected. Immunoglobulin analysis showed an elevated IgG level of 19.28 g/L, with normal complement levels.

**Figure 1:**
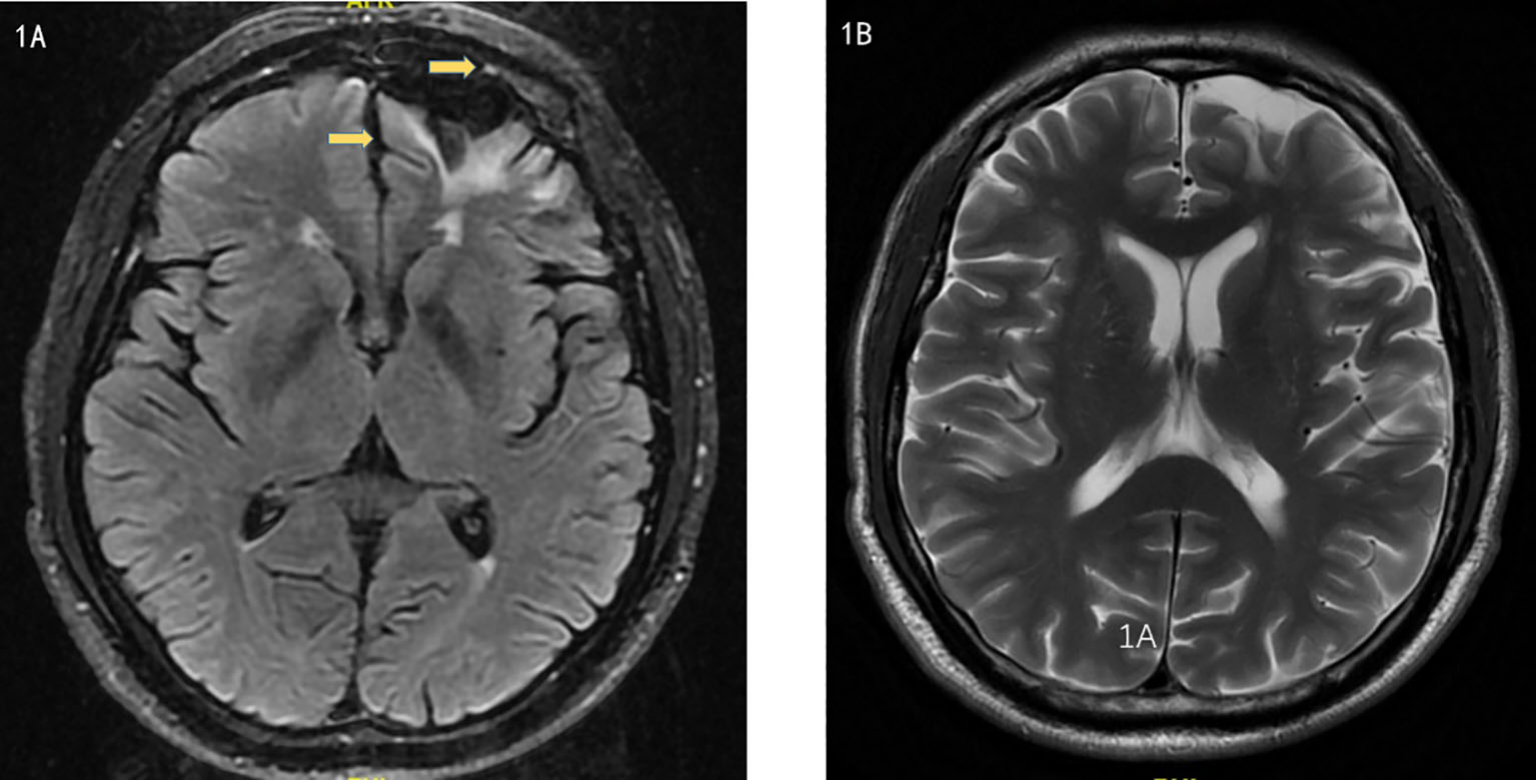
**(A, B)**. Magnetic Resonance Imaging showed multiple small ischemic lesions in the brain and a softening lesion in the left frontal lobe.

**Figure 2:**
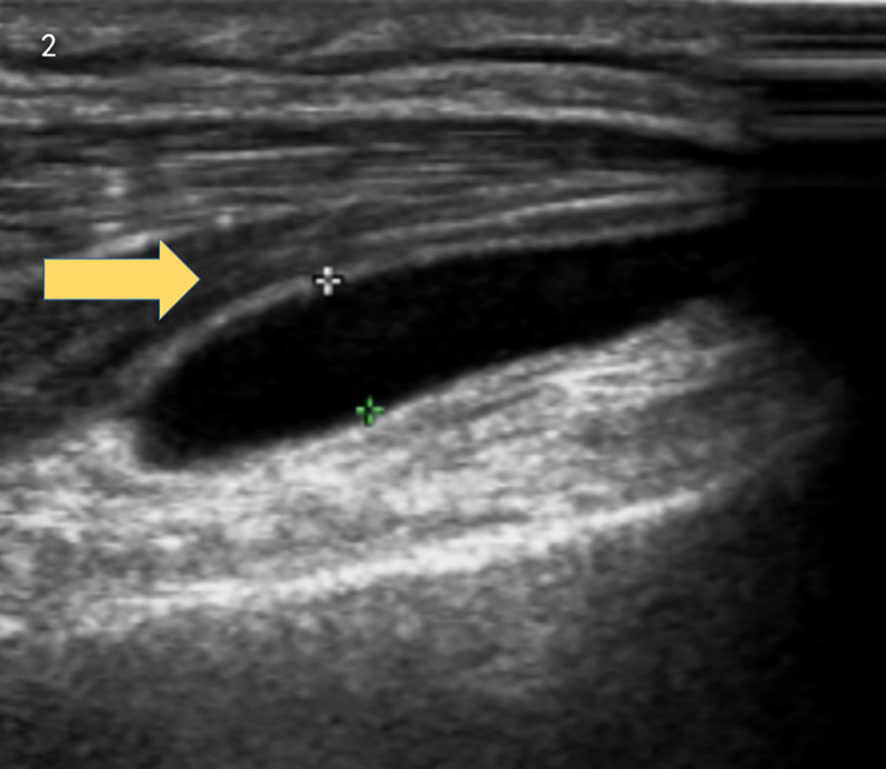
Ultrasound revealed right lateral epicondylitis and right knee joint effusion.

Brain MRI indicated multiple small ischemic lesions and a softening lesion in the left frontal lobe (**Figures 1A, B**). Imaging studies included ultrasound, revealing right lateral epicondylitis and right knee effusion (**Figure 2**). Echocardiography identified severe pulmonary hypertension and widened pulmonary arteries with findings consistent with congenital heart disease following ventricular septal defect repair.

**Figure 3:**
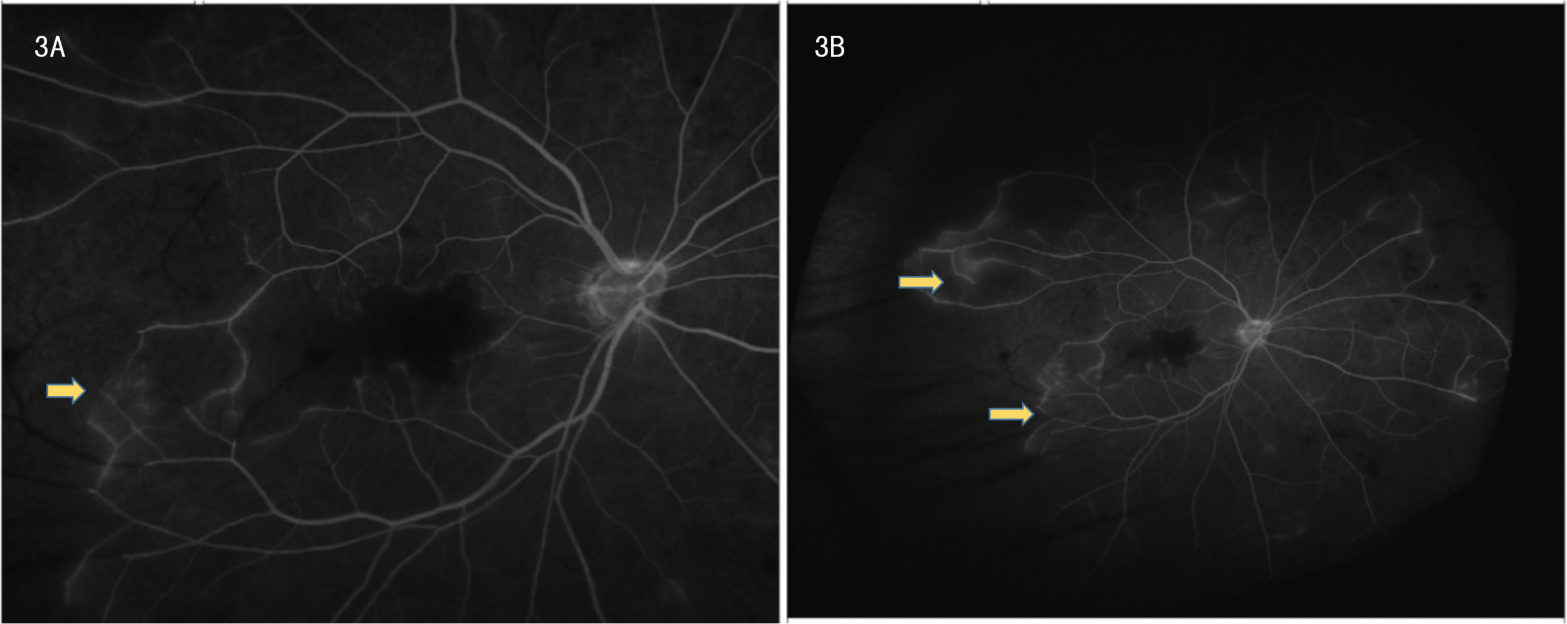
**(A, B)** Frosted branch angiitis with fern-like pattern on fundus fluorescein angiography.

Ophthalmic evaluation revealed significant bilateral vision impairment, with light perception in the right eye and hand movement in the left eye. Intraocular pressure measured 14.5 mmHg in the right eye and 14.2 mmHg in the left eye. Slit-lamp examination demonstrated bilateral clear corneas, normal anterior chambers, round pupils, and clear lenses. Ophthalmoscopy showed bilateral optic discs with normal color and margins, geographic gray-white retinal edema in the macular region, scattered ink-like hemorrhages throughout the posterior pole and peripheral retina, and segmental changes in the peripheral veins. Optical coherence tomography (OCT) confirmed bilateral macular edema, revealing hyper-reflective signals within the inner retinal layers in corresponding areas and hyper-reflective changes in the outer retina at the fovea. FFA indicated normal retinal circulation time but demonstrated extensive peripheral retinal vascular occlusion extending to the macula, along with prominent venous wall staining; these angiographic findings are consistent with occlusive vasculitis exhibiting venous predominance (**Figures 3A, B**).

**Figure 4:**
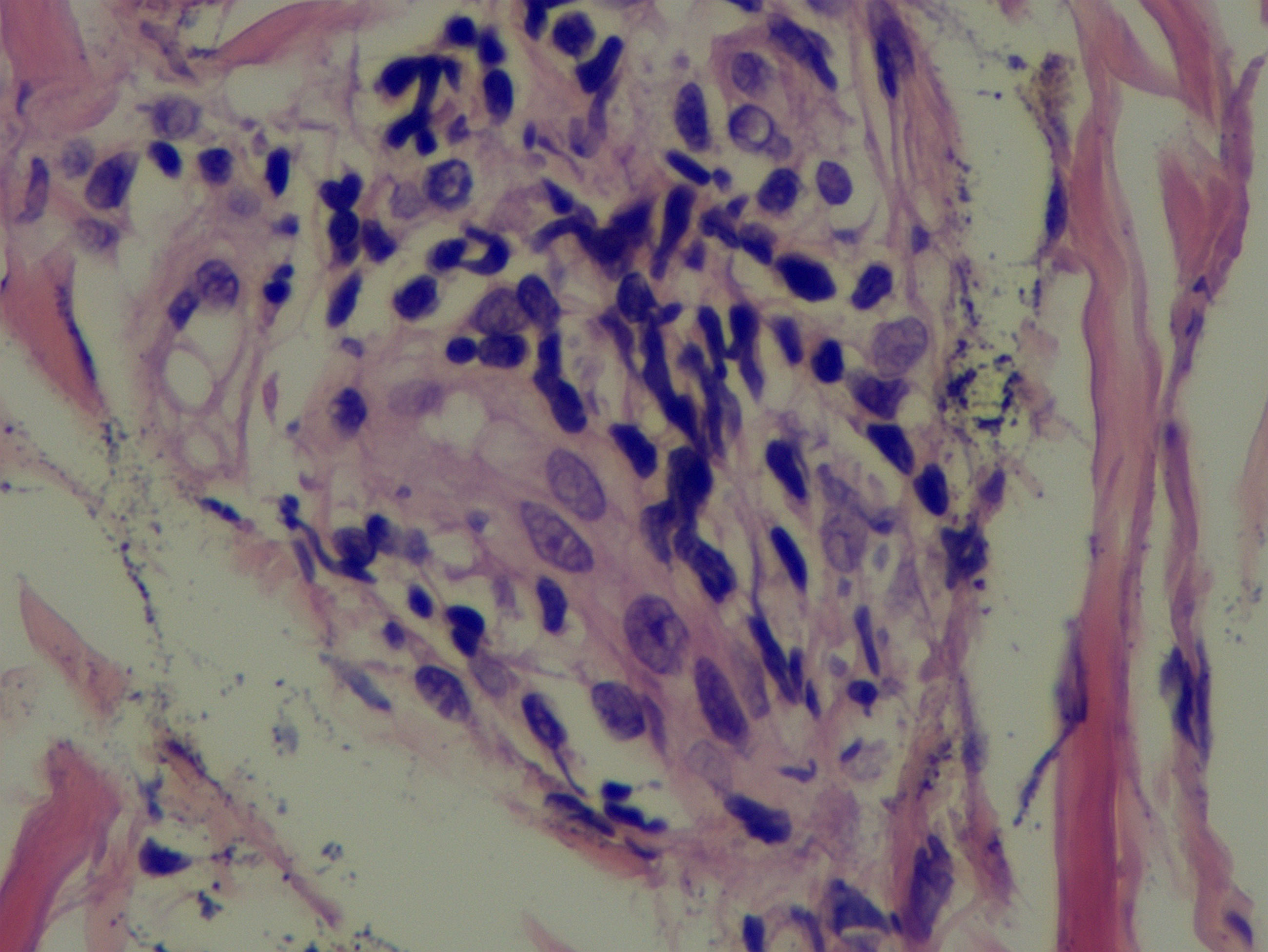
A scalp skin biopsy specimen indicated mild epidermal hyperplasia and a sparse lymphocytic infiltration, predominantly T lymphocytes, in the dermal superficial skin appendages and small blood vessels.

Scalp skin biopsy with hematoxylin and eosin staining showed mild epidermal hyperplasia, sparse lymphocytic infiltration around small vessels and skin appendages in the superficial dermis, predominantly T cells, and leukocytoclastic vasculitis. Immunohistochemical staining demonstrated CD3 (+), CD20 (–), CD30 (-), with approximately 10% of cells positive for KI-67, CD2 (+), CD5 (+), CD7 (+), CD4 (+), and a few CD8 (+) cells (**Figure 4**). The patient fulfilled the 2014 International Criteria for Behçet’s Disease (ICBD) (6), scoring 5 points derived from retinal vasculitis (2 points), recurrent oral aphthae (2 points), and folliculitis with acneiform rash (1 point), leading to the diagnosis of Behçet’s disease.

The patient initially received intravenous methylprednisolone (40 mg daily) combined with oral thalidomide (for refractory oral ulcers) and minocycline (for acneiform skin lesions), leading to partial improvement in oral ulcers and joint swelling, while acneiform lesions gradually resolved. Methylprednisolone was escalated to 500 mg intravenously daily for three days, then tapered to 40 mg daily, which resulted in complete resolution of rash and joint symptoms. Treatment with low-molecular-weight heparin and warfarin achieved partial recovery of vision and alleviation of dizziness. Long-term therapy included oral corticosteroids, cyclophosphamide, and antiviral treatment for hepatitis C virus (HCV). HCV-RNA and cryoglobulin levels before and after treatment are shown in **Table 1**. At two-year follow-up, the patient’s condition remained stable with no recurrence of major symptoms. Visual acuity partially improved: Right eye: 0.06; Left eye: 0.2.

**Table 1:** HCV-RNA and cryoglobulin levels before and after treatment.

	HCV-RNA (IU/ml)	Cryoglobulin
Before treatment	1.46×10^6	positive
After treatment	negative	negative

## Analysis and discussion

This case presents a rare and complex intersection of Behçet’s disease (BD) and hepatitis C virus (HCV)-related mixed cryoglobulinemia, manifesting with retinal vasculitis and purpuric skin lesions. While BD and cryoglobulinemia each contribute to systemic vasculitic processes, their co-occurrence is exceedingly uncommon, with few cases documented in the past several decades. This unique overlap of pathologies amplifies the inflammatory cascade, complicating both diagnosis and management.

Behçet’s disease, a chronic relapsing variable-vessel vasculitis impacting arteries and veins of all sizes, often presents with recurrent oral/genital ulcers, skin lesions, and ocular inflammation, affecting multiple systems. Its pathogenesis likely involves genetic, environmental, and immunologic factors, with HLA-B51 being a key marker linked to severe forms, particularly in East Asian and Mediterranean populations (3). Ocular involvement, especially retinal vasculitis like frosted branch angiitis (FBA), a rare clinical sign characterized by extensive perivascular sheathing and dendritic white sheaths of retinal veins, observable via funduscopy or FFA. FBA is not exclusive to BD and can occur in various infectious or autoimmune etiologies, indicates severe vascular inflammation and carries a poor prognosis if untreated (2). HLA-B51-positive BD patients are at higher risk for such severe ocular manifestations (3).

In this case, the patient’s retinal vasculitis, coupled with HLA-B51 positivity, highlights the aggressive nature of BD and underscores the need for early intervention. Despite initial treatment with corticosteroids, the persistence of visual impairment and the development of further complications necessitated a step-up in therapy, including the use of immunosuppressants like cyclophosphamide.

Cryoglobulinemia refers to the presence of cryoglobulins—immunoglobulins that precipitate at low temperatures—in the blood, typically associated with HCV infection, involves immune complexes containing rheumatoid factor and IgG that deposit in small to medium-sized vessels, leading to vasculitis. This immune complex deposition can result in systemic manifestations, including purpura, arthralgia, and peripheral neuropathy, often overlapping with BD’s own systemic effects (7, 8).

In our patient, HCV positivity and cryoglobulinemia suggest an HCV-induced immune response, further complicated by the underlying inflammatory milieu of BD. HCV infection can trigger chronic immune activation, fostering the production of cryoglobulins that exacerbate vascular inflammation. The resulting mixed cryoglobulinemic vasculitis not only contributes to the purpuric skin lesions seen in this case but may also aggravate systemic inflammation, thereby intensifying BD’s manifestations (9, 10)

It is noteworthy that cryoglobulinemia has rarely been reported in BD. The few documented cases, dating back to the 1970s, suggest that while BD primarily induces a Th1/Th17-mediated response, the concurrent HCV infection could shift this balance, promoting immune complex-mediated vasculitis. This dual pathology presents a therapeutic challenge, as treatment strategies must address both the underlying viral infection and the autoimmune vascular inflammation.

The convergence of BD and cryoglobulinemia in this patient amplifies the complexity of his condition. BD and cryoglobulinemia each involve distinct, yet overlapping, mechanisms of vasculitis. BD is primarily characterized by T-cell mediated inflammation (11–13), whereas cryoglobulinemia is driven by immune complexes, typically associated with HCV infection. In this case, the presence of both conditions likely led to an enhanced inflammatory response, with cryoglobulin deposition contributing to vascular occlusion and tissue ischemia—manifested in our patient as retinal vascular occlusion and purpuric skin lesions.

Furthermore, Behçet’s disease is a variable-vessel vasculitis capable of affecting blood vessels of any size, including both arteries and veins, combined with cryoglobulinemia’s preference for small vessels, may result in widespread vascular involvement across different organ systems. This unique interaction exemplifies how infectious agents, like HCV, can exacerbate autoimmune diseases through secondary immune responses. In managing this case, the therapeutic approach had to balance immunosuppression to control BD and cryoglobulinemia, while also addressing the underlying HCV infection to reduce cryoglobulin production.

The patient received a diagnosis of Behçet’s disease (BD) and HCV-related cryoglobulinemic vasculitis. At the time of diagnosis, the patient tested positive for hepatitis C antibody, but HCV-RNA results were still pending. Given the potential risk of high-dose corticosteroids exacerbating HCV infection—which could lead to fulminant hepatitis or hepatic failure—we initiated low-dose methylprednisolone (40 mg daily) to control the primary disease activity while minimizing the risk of viral activation.

However, due to persistent visual impairment, the corticosteroid dose was escalated to methylprednisolone 500 mg intravenously for three days, followed by a taper to 40 mg daily. Simultaneously, antiviral therapy for HCV was commenced, as viral suppression is crucial in reducing cryoglobulin production and mitigating the risk of relapse in cryoglobulinemic vasculitis. Although biologics were recommended for long-term management, the patient opted for more cost-effective traditional immunosuppressive therapy due to financial constraints. Considering the therapeutic targets for both cryoglobulinemia and refractory BD, cyclophosphamide was chosen as the preferred immunosuppressant. This approach aligns with the standard of care for cryoglobulinemia, which typically involves corticosteroids combined with either rituximab or cyclophosphamide, and is also in accordance with the treatment guidelines for refractory or moderate-to-severe BD. This multifaceted approach reflects the necessity of tailoring treatment to target both the autoimmune and viral components of the disease, thereby addressing the root cause of cryoglobulinemia while managing BD’s systemic effects (10, 14).

This case highlights several unique features that deepen our understanding of Behçet’s disease (BD) with cryoglobulinemia. The co-occurrence of BD with cryoglobulinemia-induced vasculitis is exceedingly rare, with documented cases dating back over 40 years. Managing both conditions requires careful balance of immunosuppressive therapies to control BD’s inflammatory processes while simultaneously addressing the viral etiology of cryoglobulinemia, especially in HCV-positive patients. Long-term management, as shown in this case’s two-year stable follow-up, underscores the effectiveness of a comprehensive treatment strategy targeting both autoimmune and viral factors, providing valuable insights for similar complex cases.

## Conclusion

This case exemplifies the clinical challenges posed by the rare co-occurrence of BD and HCV-related cryoglobulinemia, with retinal vasculitis as a severe manifestation. It highlights the need for a tailored, multidisciplinary approach to manage overlapping autoimmune and infectious processes. This case report contributes to the literature by expanding the understanding of BD’s interaction with secondary vasculitic conditions and underscores the importance of vigilance in identifying and managing complex vasculitic presentations in BD patients.

## Data Availability

The original contributions presented in the study are included in the article/supplementary material. Further inquiries can be directed to the corresponding author.
